# Favorable *in vitro* effects of combined IL-12 and IL-18 treatment on NK cell cytotoxicity and CD25 receptor expression in metastatic melanoma patients

**DOI:** 10.1186/s12967-015-0479-z

**Published:** 2015-04-14

**Authors:** Katarina Mirjačić Martinović, Nada Babović, Radan Džodić, Vladimir Jurišić, Suzana Matković, Gordana Konjević

**Affiliations:** Department of Experimental Oncology, Institute of Oncology and Radiology of Serbia, Pasterova 14, 11000 Belgrade, Serbia; Department of Medical Oncology, Institute of Oncology and Radiology of Serbia, Pasterova 14, 11000 Belgrade, Serbia; Surgical Oncology Clinic, Institute of Oncology and Radiology of Serbia, Pasterova 14, 11000 Belgrade, Serbia; School of Medicine, University of Belgrade, Dr Subotića 8, 11000 Beograd, Serbia; Faculty of Medical Sciences, University of Kragujevac, P.BOX 124, 34000 Kragujevac, Serbia

**Keywords:** IL-12/IL-18, Metastatic melanoma, NK cell cytotoxicity, NKG2D, CD25

## Abstract

**Background:**

As IL-12 and IL-18 have important immunostimulatory role the aim of this study was to investigate their *in vitro* effects on functional and receptor characteristics of NK cells and their subsets in healthy controls (HC) and metastatic melanoma patients (MM).

**Methods:**

Peripheral blood mononuclear cells (PBMC) of HC and MM were stimulated with culture medium alone, medium supplemented with IL-12 (10 ng/ml), IL-18 (100 ng/ml) and their combination. NK cell activity was determined using radioactive cytotoxicity assay, while perforin, CD107a and pSTAT-4 expression, IFN-γ production and the expression of NKG2D, DNAM-1, CD161, CD158a/b, CD25, IL-12R beta 1/2 receptors on CD3^−^CD56^+^ NK cells and their CD3^−^CD56^dim+^ and CD3^−^CD56^bright+^ subsets were analyzed by flow cytometry. Cytokine induced level of DAP10 in PBMC was analyzed by reverse transcription polymerase chain reaction.

**Results:**

IL-12 alone or in combination with IL-18 significantly induced NK cell activity and CD107a degranulation marker expression in MM and HC, while IL-18 alone did not have any effect in patients. The combination of IL-12 and IL-18 significantly increased mean fluorescence intensity (MFI) of IFN-γ in all NK cell subsets in HC and only in the bright subset in MM. MM that belong to M1c group with metastasis in liver and increased LDH serum values had significantly lower increase in NK cell cytotoxicity after combined IL-12 and IL-18 treatment compared to the patients in M1a and M1b categories. These results could be explained by decreased IL-12R expression and lower increase in pSTAT-4 and perforin expression in NK cells of M1c patients after IL-12 and combined IL-12 and IL-18 treatment. IL-18 alone significantly decreased NKG2D receptor expression and level of DAP10 signaling molecule in MM, while combined IL-12 and IL-18 increased the expression of CD25 on all NK cell subsets in HC and MM. Additionally, MM that belong to M1a + M1b group had significantly higher increase in CD25 receptor expression compared to the patients in M1c group.

**Conclusions:**

The novel data obtained in this study support the use of IL-12 and IL-18 in combination for developing new therapeutic strategies for metastatic melanoma especially for patients with better survival rate and prognosis.

## Background

Human natural killer (NK) cells are important components of the innate immune system that are able to lyse transformed cells without prior sensitization. They exert cytotoxic function by releasing perforin and granzymes from their granules and have an immunoregulatory role by producing many cytokines especially IFN-γ that regulate adaptive T-cell-mediated anti-tumor response [1]. CD107a, the main membrane molecule of cytolytic granules of NK cells is strongly upregulated on the surface of these cells after their contact with tumor and its expression is associated with perforin release. In this sense, CD107a has been described as a marker of NK cell degranulation and cytotoxicity [2].

The balance between NK cell activating and inhibitory signals mediated by various receptors regulates NK cell effector functions [3]. The most important activating NK receptors are NKG2D, natural cytotoxicity receptors (NCR) and DNAX accessory molecule-1 (DNAM-1). NKG2D, as the most prominent activating c-lectin-like receptor, mediates immune responses that are beneficial in the surveillance against cancer [4]. This receptor upon binding to stress induced ligands constitutively expressed on transformed cells such as MHC-class-I-related molecules, MICA/MICB and ULBP1-4, induces NK cell cytotoxicity in association with DAP10 adaptor molecule [5]. DNAM-1 is a co-receptor expressed by virtually all NK cells and its stimulation by interaction with the members of the nectin family, PVR (CD155) and Nectin-2 (CD112), on tumor cells leads to NK cell activation and target cell lysis [6]. Another common NK cell receptor, CD161, one of the earliest markers in NK cell development, is primarily described as an activating receptor [7].

Contrary to this, killer immunoglobulin-like receptors (KIRs) that belong to the immunoglobulin superfamily are responsible for the inhibition of NK cell-mediated lysis of normal cells that express MHC-I molecules. In this sense, according to the “missing-self” hypothesis, the activation of NK cells occurs in contact with malignantly transformed cells that have lost MHC-I molecules, and therefore become susceptible to lysis [8]. According to the length of their cytoplasmatic tail KIR are classified into long (e.g., KIR2DL and KIR3DL), inhibitory, and short (e.g., KIR2DS and KIR3DS) stimulating receptors, although inhibitory KIRs are dominant [9].

In humans, CD3^−^CD56^+^ NK cells can be subdivided in two functionally and phenotipically different subsets based on CD56 receptor expression. CD3^−^CD56^dim+^ subset, that aside from high expression of inhibitory KIR receptors and CD16 activating receptor, also expresses abundant perforin and granzymes in their granules and is involved in NK cell cytotoxicity. The other CD3^−^CD56^bright+^ subset, with low expression of CD16 and low to absent expression of KIR receptors has a regulatory function, owing to its abundant cytokine production and weak cytotoxicity [10].

As it is known that melanoma patients with advanced disease have impairments in their immune response, including decreased NK cell activity [11], and that melanoma is an immunogenic tumor resistant to chemotherapy and irradiation, immunomodulating agents such as cytokines have been included in its treatment [12]. In this sense, IFN-α and IL-2 are administrated in the immunotherapy of melanoma for several decades [12,13]. On the other hand, considering that their therapeutic effects are limited, many other cytokines, such as IL-12 and IL-18 have been investigated in this disease [14,15].

Interleukin-12 (IL-12) is a cytokine that is produced primarily by antigen-presenting cells and by enhancing NK and T cell cytotoxicity, inducing IFN-γ production from these cells and favoriting Th1 differentiation plays an essential role in the interaction between the innate and adaptive immunity [16]. Interleukin-18 (IL-18) is an immunoregulatory and proinflammatory cytokine that belong to the IL-1 family. It shows numerous effects not only on NK cells, but also on monocytes, dendritic cells (DCs), T cells. IL-18 acts synergistically with IL-12 to promote cytotoxicity and IFN-γ production from NK and T cells [17] and also it is involved in NK cell priming [18] and the interaction between DCs and NK cells [19].

As IL-12 and IL-18 have an important role in improving anti-tumor immune response they could have a great potential in cancer immunotherapy. We, therefore, consider that it is of interest to assess *in vitro* effects of IL-12, IL-18 and their combination on NK cell effector functions, cytotoxicity and IFN-γ production, as well as on the expression of numerous receptors on NK cells and their dim and bright subsets in metastatic melanoma (MM) patients and healthy controls (HC).

## Methods

### Blood samples

Peripheral venous blood was obtained from 36 MM patients (stage IV according to 7^th^ modified AJCC/UICC staging system) [20] and 26 HC, age and gender matched, with no evidence of any disease or infection. Blood was drawn at the time of diagnosis prior to chemotherapy. Before inclusion in the study, informed consent was signed by each patient and healthy volunteer and approved by the Ethical committee of Institute of Oncology and Radiology of Serbia. The characteristics of MM patients and HC enrolled in this study are listed in Table 1. Furthermore, MM patients are divided in 2 groups based on the localization of distant metastases according to AJCC/UICC staging system. Patients that have metastases in distant skin, the subcutaneous layer or in distant lymph nodes and normal values of serum lactate dehydrogenase (LDH) (<460 IU/l) (M1a) and patients with metastases in the lungs (M1b) are included in M1a + M1b group, while the patients with metastases in vital organs other than the lungs with normal serum LDH level or the patients that have any distant metastasis with elevated LDH (>460 IU/l) are included in M1c group.

**Table 1:** **The characteristics of metastatic melanoma (MM) patients and healthy controls (HC)**

		**MM**	**HC**
		**n = 26**	**n = 36**
**Age:**			
	**Range**	36-67	37-73
	**Median**	51	54
**Gender:**			
	**Male**	14	17
	**Female**	12	19
**Metastases:**			
	**Distant skin**		10
	**Subcutaneous layer**		7
	**Distant lymph nodes:**		16
	**axillary**		8
	**inguinaly**		7
	**retroperitonealy**		1
	**Lungs**		10
	**Liver**		10
	**Spleen**		3
	**Suprarenal gland**		4
	**Bones**		3
**LDH serum values:**			
	**normal (<460 IU/l)**		20
	**increased (>460 IU/l)**		16
**Classification of distant metastases:**			
	**M1a**		11
	**M1b**		7
	**M1c**		18

### Peripheral blood mononuclear cell (PBMC) isolation

PBMC were isolated from heparinized blood obtained from HC and MM patients using Lymphoprep (Nypacon, Oslo, Norway) density gradient, centrifuged at 500 g for 40 min, and washed three times in RPMI 1640 culture medium (CM), (Sigma, St. Louis, USA) supplemented with 10% fetal calf serum (FCS) (Sigma). After washing PBMC were immediately used for functional, phenotypic and molecular analysis.

### *In vitro* treatment of PBMC with various cytokines

PBMC isolated from HC and MM patients were cultivated in CM alone, CM supplemented with IL-12 (10 ng/ml) (Becton Dickinson, San Jose, USA), IL-18 (100 ng/ml) (R&D, Minneapolis, USA) and IL-12 and IL-18 in combination, in six well plates at 37°C and 5% CO2 in humid atmosphere.

### NK cell assay

PBMC was determined using standard cytotoxicity assay [21]. One hundred microlitres of PBMC, as effector cells, at concentration of 4.0 × 10^6^/ml of CM and two 1:1 dilutions were mixed with 100 μl of the erythromyeloid cell line K562, as target cells, at concentration of 0.05 × 10^6^/ml, prelabeled with radioactive ^51^Chromium (Na2CrO4, As = 3.7 MBq, Amersham, UK), to form triplicates of three effector cell (E) to target cell (T) ratios (E:T), 80:1, 40:1 and 20:1. The assay was performed in 96 round bottom microwell plates (Sigma), which were incubated in an incubator at 37°C, in a humidified atmosphere containing 5% CO_2_. Plates were, then centrifuged for 3 min at 200 g and the supernatant from each well was used for determination of the amount of released ^51^Chromium from the lysed K562 cells in a gamma counter (Berthold, Oak Ridge, USA) and expressed in counts per minute (cpm). The mean percent cytotoxicity was calculated using the following formula:

$$ \frac{\mathrm{cpm}\ \left(\mathrm{experimental}\ \mathrm{release}\right)\hbox{-} \mathrm{cpm}\ \left(\mathrm{spontaneous}\ \mathrm{release}\right)}{\mathrm{cpm}\ \left(\mathrm{maximal}\ \mathrm{release}\right)\ \hbox{-}\ \mathrm{cpm}\ \left(\mathrm{spontaneous}\ \mathrm{release}\right)}\kern0.5em \mathrm{X}\ 100 $$

Maximal release was obtained by incubation of target K562 tumor cells at the same concentration in the presence of 5% TritonX-100, and spontaneous release was obtained by incubation of K562 cells in culture medium, alone.

### Flow cytometric analysis

Surface immuno-phenotype of 18 h *in vitro* treated PBMC subsets were identified using the following combinations of directly labeled monoclonal antibodies (mAbs): CD3PerCP (clone SK7)/CD56FITC (clone NCAM 16.2)/IL-12Rbeta1PE (clone 2.4E6), CD3PerCP/CD56FITC/IL-12Rbeta2PE (clone 2B6/12 beta 2), CD3PerCP/CD56FITC/DNAM-1PE (clone DX11), CD3PerCP/CD56FITC/CD161PE (clone DX12), CD3PerCP/CD56FITC/CD158bPE (clone CH-L), CD3PerCP/CD56PE (clone NCAM 16.2)/CD158aFITC (clone HP-3E4), CD3PerCP/CD56FITC/CD25PE (clone M-A251) (Becton Dickinson) and CD3PerCP/CD56FITC/NKG2DPE (clone 149810) (R&D). The samples were prepared as previously described [22]. Briefly, 1.0 × 10^5^ PBMC in 100 μl RPMI 1640 supplemented with 10% FCS were incubated for 30 min at 4°C with 20 μl of appropriate mAbs combination, washed twice with ice-cold phosphate-buffered saline (PBS) and fixed with 1% paraformaldehyde prior to flow cytometry analysis. Surface marker expression was quantified on FACSCalibur flow cytometer (Becton Dickinson). A total of 50 000 gated events, verified as PBL according to their physical characteristics (FSC and SSC), were collected per sample and analyzed using CellQUEST software. Exclusion of non-specific fluorescence was based on matched IgG1 isotype mAb combinations conjugated with FITC (clone X39), PE (clone X40) and PerCP (clone SK7) (Becton Dickinson). NK cells were defined and gated within the lymphocyte gate according to their expression of CD3 and CD56 (CD3^−^CD56^+^). In order to define the two NK cell subsets of CD56 low, i.e., CD3^−^CD56^dim+^ or CD56 high, i.e., CD3^−^CD56^bright+^ subsets, CD3^−^CD56^+^ NK cells were divided based on the density of CD56 antigen defined by mean fluorescence intensity (MFI). MFI refers to the fluorescence intensity of each event in average and represents the expression quantity of the parameter on each event. NK cell receptors, IL-12R beta 1, IL-12R beta 2, NKG2D, DNAM-1, CD161, CD158a, CD158b and CD25 were expressed as the percentage or MFI on gated CD3^−^CD56^+^ NK cells and their CD3^−^CD56^dim+^ and CD3^−^CD56^bright+^ NK cell subsets.

### CD 107 degranulation assay

100 μl of 18 h *in vitro* stimulated PBMC (3x10^6^cells/ml) from HC and MM patients were cultured with 100 μl of washed K562 (2x10^6^/ml) for 4 hours at 37°C in a humidified atmosphere in CO_2_ incubator. Monensin (Sigma) at the final concentration of 2 μl/ml was added after 1 hour of incubation. Cells were afterwards stained with CD56FITC, CD107aPE (clone H4A3) and CD3PerCP mAbs (Becton Dickinson). The percentage and MFI of CD107a was estimated on gated CD3^−^CD56^+^ NK cells, as well as on CD3^−^CD56^dim+^ and CD3^−^CD56^bright+^ NK subsets by flow cytometry. Spontaneous CD107a expression was determined in absence of K562 cells.

### Intracellular IFN-gamma, phosphorylated STAT-4 and perforin analyses

For measurement of phosphorylated pSTAT-4 20 min and for measurement of IFN-γ and perforin 18 h *in vitro* stimulated PBMC (1x10^6^/ml) from HC and MM patients were analyzed. Three hours before IFN-γ analysis, brefeldin A was added at concentration of 10 μg/ml. The cells were then stained with mAbs against cell surface molecules: CD3PerCP, CD56PE (for IFN-γ analysis) and CD56FITC (for pSTAT-4 and perforin analyses) (Becton Dickinson), followed by fixing and permeabilizing with the Permeabilizing Solution 2 (Perm 2) (Becton Dickinson) accords to the manufacturer’s instructions. Then the cells were incubated with anti-IFN-γFITC (clone 25723.11), anti-pSTAT-4PE (clone 38/p-Stata4) (Becton Dickinson), anti-Perforin RPE (clone deltaG9) (Invitrogen, Madison, USA) or IgG1 isotype control (Becton Dickinson). Finally, the cells were washed and analyzed by flow cytometry directly after preparation. The percentages and MFI of intracellular IFN-γ, pSTAT-4 and perforin were analyzed in CD3^−^CD56^+^ NK cells and their CD3^−^CD56^dim+^ and CD3^−^CD56^bright+^ subsets.

### RT-PCR

For the analysis of DAP10 transcription level total RNA was isolated from 18 h *in vitro* stimulated PBMC of HC and MM patients using Trizol reagent (Invitrogen). One microgram of total RNA was reverse transcribed by random priming, incubated with 1 μl reverse transcriptase MuLV (Fermentas, St. Leon Rot, Germany), 10 mM deoxynucleotide triphosphate mix (dNTP) (Fermentas), RNAse inhibitor (Fermentas) and 2 μl 0.1 M dithiothreitol (DTT) (Fermentas) for 1 h at 37°C and then 5 min at 99°C. Total cDNA was then amplified by PCR with specific primers: DAP-10 (350 bp) (up: 5′-CAG ACC CCA GTC CAC CAT G-3′ and down: 5′-GTG CCA CCA CAC ACC ATC-3′) and beta-Actin as housekeeping gene (685 bp) (sense: 5′- TGG GTC AGA AGG ATT CCT AT-3′ and antisense: 5′- AAG GAA GGC TGG AAG AGT -3′). The optimal cycle conditions were 30 sec at 94°C, 30 sec at 55°C, 30 sec at 72°C for 30 cycles. RT-PCR results were analyzed on 1.5% agarose gels.

### Quantification of blots and gels

Agarose gels were scanned by using gel-image system (Kodak Image 1D Image 1 3.6.), in a grey scale mode at 169 mm pixel size and 1250–1650 (X–Y) pixel count, using the autodensity feature on a scale ranging from 0 (clear) to 255 (opaque). The pixel density was determined and used to calculate the integrated density of a selected band. Values of integrated density were reported in volume units of pixel intensity per mm^2^.

### Statistical analysis

Significance of differences for obtained results was done by nonparametric Wilcoxon signed rank test and Mann–Whitney exact test.

## Results

### The effect of investigated cytokines on NK cell cytotoxic activity and IFN-γ production

**Figure 1:**
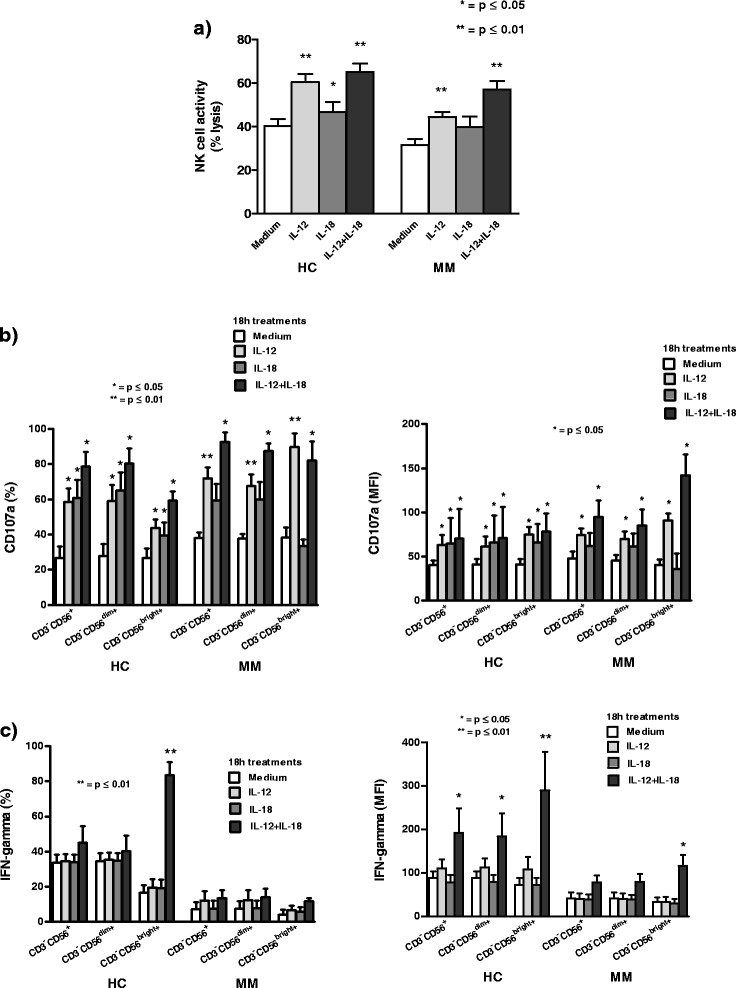
The effect of cytokines on NK cell cytotoxic activity and IFN-γ production. **a)** In healthy controls (HC) and metastatic melanoma (MM) patients high significant (**p < 0.01, Wilcoxon signed rank test) enhancement of NK cell cytotoxicity is obtained after 18 h *in vitro* treatment of peripheral blood mononuclear cells (PBMC) with IL-12 alone (10 ng/ml), as well as with IL-12 in combination with IL-18 (100 ng/ml) that gives a significant (*p < 0.05, Wilcoxon signed rank test) increase in HC only; **b)** In both HC and MM patients IL-12 alone or in combination with IL-18 significantly (*p < 0.05, Wilcoxon signed rank test) or high significantly (**p < 0.01, Wilcoxon signed rank test) increases the percentage and mean fluorescence intensity (MFI) of CD107a degranulation marker on CD3^−^CD56^+^ NK cells and their CD3^−^CD56^dim+^ and CD3^−^CD56^bright+^ subsets, while IL-18 alone significantly (*p < 0.05, Wilcoxon signed rank test) increases the expression of CD107a on NK cells and their subsets only in HC. Results are obtained by Flow cytometry; **c)** In HC 18 h *in vitro* treatment of PBMC with combination of IL-12 and IL-18 high significantly (**p < 0.01, Wilcoxon signed rank test) increases the percentage of IFN-γ only in the bright NK cell subset, while this combined cytokine treatment increases the MFI of IFN-γ significantly (*p < 0.05, Wilcoxon signed rank test) in NK cells and their dim subset and high significantly (**p < 0.01, Wilcoxon signed rank test) in the bright NK cell subset. In MM patients the combination of IL-12 and IL-18 significantly (*p < 0.05, Wilcoxon signed rank test) increases only the MFI of IFN-γ in the bright subset. Results are obtained by Flow cytometry. All results are shown as mean ± SE for maximum 26 HC and 36 MM patients.

We show that in both HC and MM patients NK cell cytotoxic activity performed against standard sensitive erythromyeloid K562 tumor target cell line after treatment with 10 ng/ml of IL-12 alone or in combination with IL-18 (100 ng/ml) is high significantly (p < 0.01, Wilcoxon signed rank test) enhanced, while after treatment with IL-18 alone is significantly (p < 0.05, Wilcoxon signed rank test) augmented only in HC compared to the results in CM. Contrary to this, in investigated MM patients IL-18 alone does not significantly (p > 0.05, Wilcoxon signed rank test) potentiate NK cell activity (Figure 1a).

Our results also indicate that in both HC and MM patients IL-12 alone or in combination with IL-18 significantly (p < 0.05, Wilcoxon signed rank test) or high significantly (p < 0.01, Wilcoxon signed rank test) increases the percentage or MFI of CD107a degranulation marker on NK cells and both their dim and bright subsets. Contrary to this, IL-18 alone, compared to the results in CM, significantly (p < 0.05, Wilcoxon signed rank test) increases the expression of CD107a only in HC (Figure 1b).

Furthermore, we analyze the production of intracellular IFN-γ in NK cells and their dim and bright subsets in HC and MM patients after 18 h PBMC *in vitro* treatments with IL-12, IL-18 and their combination. We show that in HC, combined IL-12 and IL-18 treatment high significantly (p < 0.01, Wilcoxon signed rank test) increases the percentage of IFN-γ only in the bright NK cell subset, while this cytokine treatment increases the MFI of IFN-γ significantly (p < 0.05, Wilcoxon signed rank test) in NK cells and the dim subset and high significantly (p < 0.01, Wilcoxon signed rank test) in the bright NK cell subset. In MM patients the combination of IL-12 and IL-18 significantly (p < 0.05, Wilcoxon signed rank test) increases only the MFI of IFN-γ in the bright subset. Contrary to this, IL-12 or IL-18 alone does not have any significant (p > 0.05, Wilcoxon signed rank test) effect on IFN-γ production in NK cells and both their subsets in HC, as well as in MM patients (Figure 1c).

### The effect of investigated cytokines on the percentages and absolute values of NK cells and their subsets

All investigated cytokines do not have any significant effect (p > 0.05, Wilcoxon signed rank test) on the percentages, as well as on the absolute values of NK cells and their dim and bright subsets in HC and MM patients (Table 2).

**Table 2:** **The percentages and absolute values of NK cell subsets after cytokine** ***in vitro*** **treatment**

		**18 h** ***in vitro*** **treatment**				
			**Medium**	**IL-12**	**IL-18**	**IL-12 + IL-18**
**Controls (n = 26)**	**Percentage (%)**	**CD3** ^**−**^ **CD56** ^**+**^	13.76 ± 1.75^#^	14.58 ± 2.67	13.92 ± 2.66	12.47 ± 2.14
		**CD3** ^**−**^ **CD56** ^**dim+**^	12.72 ± 1.76	13.39 ± 2.64	12.04 ± 2.26	11.53 ± 2.13
		**CD3** ^**−**^ **CD56** ^**bright+**^	1.06 ± 0.11	1.18 ± 0.15	1.07 ± 0.20	0.90 ± 0.12
	**Apsolute values (/μl)**	**CD3** ^**−**^ **CD56** ^**+**^	261.53 ± 19.33	280.53 ± 29.33	255.28 ± 21.38	275.14 ± 23.33
		**CD3** ^**−**^ **CD56** ^**dim+**^	250.87 ± 30.54	260.71 ± 32.55	249.39 ± 33.83	259.12 ± 35.22
		**CD3** ^**−**^ **CD56** ^**bright+**^	19.63 ± 1.39	21.13 ± 0.19	18.87 ± 0.09	20.18 ± 1.01
**Patients (n = 36)**	**Percentage (%)**	**CD3** ^**−**^ **CD56** ^**+**^	16.61 ± 1.99	16.82 ± 2.11	17.70 ± 2.42	17.72 ± 2.31
		**CD3** ^**−**^ **CD56** ^**dim+**^	15.56 ± 1.96	15.82 ± 2.10	16.63 ± 2.40	16.66 ± 2.29
		**CD3** ^**−**^ **CD56** ^**bright+**^	1.05 ± 0.09	1.06 ± 0.08	1.07 ± 0.10	1.03 ± 0.12
	**Apsolute values (/μl)**	**CD3** ^**−**^ **CD56** ^**+**^	328.32 ± 21.33	319.83 ± 28.33	319.83 ± 25.13	338.51 ± 23.33
		**CD3** ^**−**^ **CD56** ^**dim+**^	305.13 ± 20.96	300.75 ± 39.73	321.27 ± 27.23	325.17 ± 27.71
		**CD3** ^**−**^ **CD56** ^**bright+**^	22.87 ± 0.19	25.34 ± 0.09	26.13 ± 0.33	20.01 ± 0.10

#Results are expressed as mean values ± SE. Absolute numbers were calculated by multiplying PBL number in the blood count with percentage for each subset obtained by flow cytometry.

### The differences in cytokine-induced increase in NK cell activity between M1a + M1b and M1c metastatic melanoma patients

**Figure 2:**
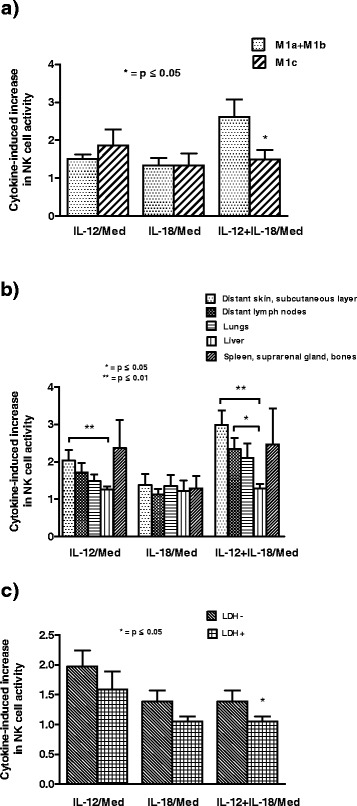
Cytokine-induced increase in NK cell activity in M1a + M1b and M1c metastatic melanoma patients. **a)** Patients in M1c group have significantly (*p < 0.05, Mann–Whitney exact test) lower enhancement in NK cell cytotoxicity after *in vitro* treatment with combination of IL-12 and IL-18 compared to the increase in M1a + M1b group; **b) **Metastatic melanoma (MM) patients that have distant metastasis in liver have significantly (*p < 0.05, Mann–Whitney exact test) or high significantly (**p < 0.01, Mann–Whitney exact test) lower enhancement in NK cell cytotoxicity after *in vitro* treatment with IL-12 alone or in combination with IL-18 compared to the increase in NK cell cytotoxicity in MM patients with metastases in skin, subcutaneous layer and lymph nodes; **c) **MM patients with increased values of serum lactate dehydrogenase (LDH+) have significantly lower (*p < 0.05, Mann–Whitney exact test) upregulation of NK cell cytotoxicity after combined IL-12 and IL-18 *in vitro* treatment compared to patients with normal LDH values (LDH-). Results are expressed in indexes calculated as the value of NK cell cytotoxicity after the cytokine treatment of each MM patient devides with the value of NK cell cytotoxicity after treatment in medium alone. All results are shown as mean ± SE for maximum 36 MM patients.

We show that patients in M1c group have significantly (p < 0.05, Mann–Whitney exact test) lower enhancement in NK cell cytotoxicity after *in vitro* treatment with combination of IL-12 and IL-18 compared to the increase in M1a + M1b group. IL-12 or IL-18 alone has the similar effect on the induction of NK activity in both groups of investigated MM patients (Figure 2a).

Furthermore, MM patients that have distant metastasis in liver have significantly (p < 0.05, Mann–Whitney exact test) or high significantly (p < 0.01, Mann–Whitney exact test) lower enhancement in NK cell cytotoxicity after *in vitro* treatment with IL-12 alone or in combination with IL-18 compared to the increase in NK cell cytotoxicity in MM patients with distant metastases in skin, subcutaneous layer and lymph nodes. IL-18 alone has the similar effect on the induction of NK activity in all investigated groups of MM patients (Figure 2b).

MM patients with increased values of serum LDH (LDH+) have significantly lower (p < 0.05, Mann–Whitney exact test) upregulation of NK cell cytotoxicity after combined IL-12 and IL-18 *in vitro* treatment compared to patients with normal LDH values (LDH-) (Figure 2c).

### The differences in IL-12 receptor expression and cytokine-induced increase in pSTAT-4 and perforin expression in NK cells between M1a + M1b and M1c metastatic melanoma patients

**Figure 3:**
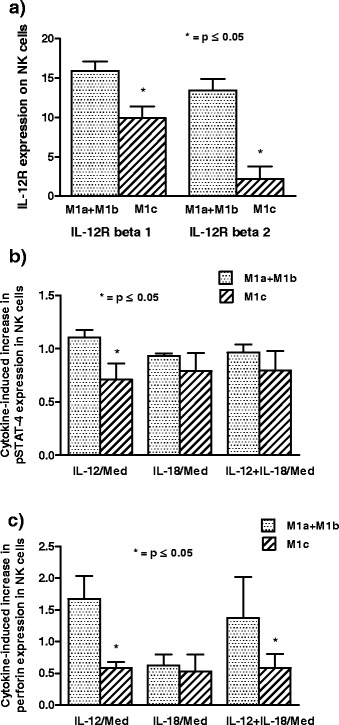
IL-12 receptor and cytokine-induced increase in pSTAT-4 and perforin expression in M1a + M1b and M1c patients. **a)** Metastatic melanoma (MM) patients in M1c group have significantly (*p < 0.05, Mann–Whitney exact test) lower expression of IL-12R beta 1 and IL-12R beta 2 on their NK cells compared to MM patients that belong to M1a + M1b group. Results are obtained by Flow cytometry; **b)** 20 min *in vitro* treatment with IL-12 increases pSTAT-4 expression in NK cells significantly (*p < 0.05, Mann–Whitney exact test) lower in M1c group of patients compared to the increase in M1a + M1b group; **c)** Mean fluorescence intensity (MFI) of perforin in NK cells is significantly (*p < 0.05, Mann–Whitney exact test) lower upregulated after *in vitro* treatment with IL-12 alone or in combination with IL-18 in M1c group of patients compared to M1a + M1b group. Results are obtained by Flow cytometry and are expressed in indexes calculated as the value of pSTAT-4 or perforin after the cytokine treatment of each MM patient devides with the value of appropriate parameter after treatment in medium alone. All results are shown as mean ± SE for 20 MM patients.

Furthermore, we show that MM patients that belong to M1c group have significantly (p < 0.05, Mann–Whitney exact test) lower expression of both IL-12R, IL-12R beta 1 and IL-12R beta 2, on their NK cells compared to patients in M1a + M1b group (Figure 3a).

Analyzing the influence of the investigated cytokines on pSTAT-4 signaling molecule expression in NK cells of MM patients by Flow cytometry we show that 20 min *in vitro* treatment with IL-12 increases this molecule MFI significantly (p < 0.05, Mann–Whitney exact test) lower in M1c group of patients compared to the increase in M1a + M1b group. IL-18 alone or in combination with IL-12 has the similar effect (p > 0.05, Wilcoxon signed rank test) on the induction of pSTAT-4 expression in NK cells in both groups of investigated MM patients (Figure 3b).

MFI of cytotoxic mediator, perforin, in NK cells is significantly (p < 0.05, Mann–Whitney exact test) lower upregulated after *in vitro* treatment with IL-12 alone or in combination with IL-18 in M1c group of patients compared to M1a + M1b group. Contrary to this, IL-18 alone has the similar effect on the induction of perforin expression in NK cells in both groups of patients (p > 0.05, Wilcoxon signed rank test) (Figure 3c).

### The effect of investigated cytokines on the activating NKG2D, DNAM-1 and CD161 receptor expression on NK cells and their subsets

**Figure 4:**
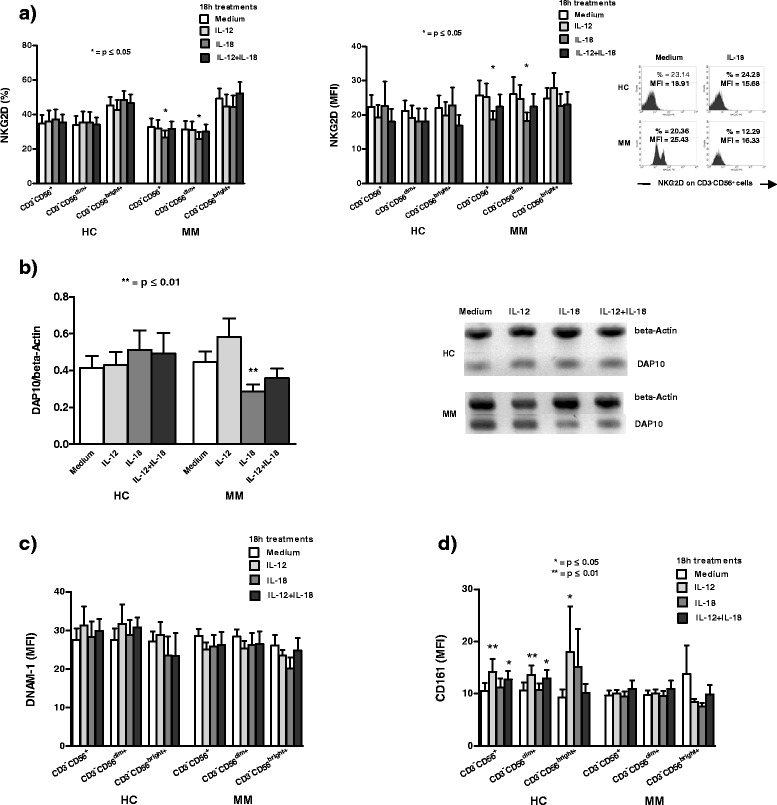
The effect of cytokines on NKG2D, DNAM-1 and CD161 receptor expression on NK cell subsets. **a)** Percentage and mean fluorescence intensity (MFI) of NKG2D receptor decrease significantly (*p < 0.05, Wilcoxon signed rank test) on CD3^−^CD56^+^ NK cells, as well as on CD3^−^CD56^dim+^ subset in metastatic melanoma (MM) patients after 18 h *in vitro* treatment with IL-18. Results are obtained by Flow cytometry; **b)** 18 h *in vitro* treatment with IL-18 high significantly (**p < 0.01, exact Wilcoxon signed rank test) decreases DAP10 mRNA level in peripheral blood mononuclear cells (PBMC) of MM patients. Results are obtained by RT-PCR with respect to β-actin level; **c)** MFI of DNAM-1 co-receptor does not change significantly (p > 0.05, Wilcoxon signed rank test) on NK cells and both their subsets in healthy controls (HC) and MM patients after 18 h *in vitro* treatments of PBMC with investigated cytokines. Results are obtained by Flow cytometry; **d)** In HC IL-12 alone or in combination with IL-18 significantly (*p < 0.05, Wilcoxon signed rank test) or high significantly (**p < 0.01, Wilcoxon signed rank test) increases MFI of CD161 receptor on NK cells and their subsets. Results are obtained by Flow cytometry. All results are expressed as mean ± SE for 19 HC and 23 MM patients.

Analyzing the modulation of NKG2D activating receptor expression on CD3^−^CD56^+^ NK cells and their CD3^−^CD56^dim+^ and CD3^−^CD56^bright+^ subsets with all investigated cytokines we show that both percentage and MFI of this receptor decrease significantly (p < 0.05, Wilcoxon signed rank test) only in MM patients after 18 h *in vitro* treatment with IL-18 on NK cells, as well as on their dim subset. IL-12 alone or in combination with IL-18 does not have any significant (p > 0.05, Wilcoxon signed rank test) effect on the expression of this NK cell receptor on NK cells, as well as on their subsets in HC and in MM patients (Figure 4a).

Analyzing the modulation of DAP10, NKG2D adaptor molecule, after 18 h cytokine treatments of PBMC in HC, as well as in MM patients, we show that IL-18 alone high significantly (p < 0.01, exact Wilcoxon signed rank test) decreases mRNA level of this signaling molecule only in MM patients (Figure 4b).

Analyzing the expression of DNAM-1 on NK cells and their subsets, we show that, compared to treatment in CM, the MFI of this receptor does not change significantly (p > 0.05, Wilcoxon signed rank test) after 18 h *in vitro* treatments with all investigated cytokines in HC, as well as in MM patients (Figure 4c).

Obtained results from activating NK cell receptor, CD161, show that in HC IL-12 alone or in combination with IL-18 significantly (p < 0.05, Wilcoxon signed rank test) or high significantly (p < 0.01, Wilcoxon signed rank test) increases MFI of this receptor on NK cells and their subsets. Contrary to this, in MM patients the investigated cytokines do not have any significant (p > 0.05, Wilcoxon signed rank test) effect on CD161 expression on NK cells and both dim and bright subsets (Figure 4d).

### The effect of investigated cytokines on the inhibitory KIR, CD158a and CD158b, receptor expression on NK cells and their subsets

**Figure 5:**
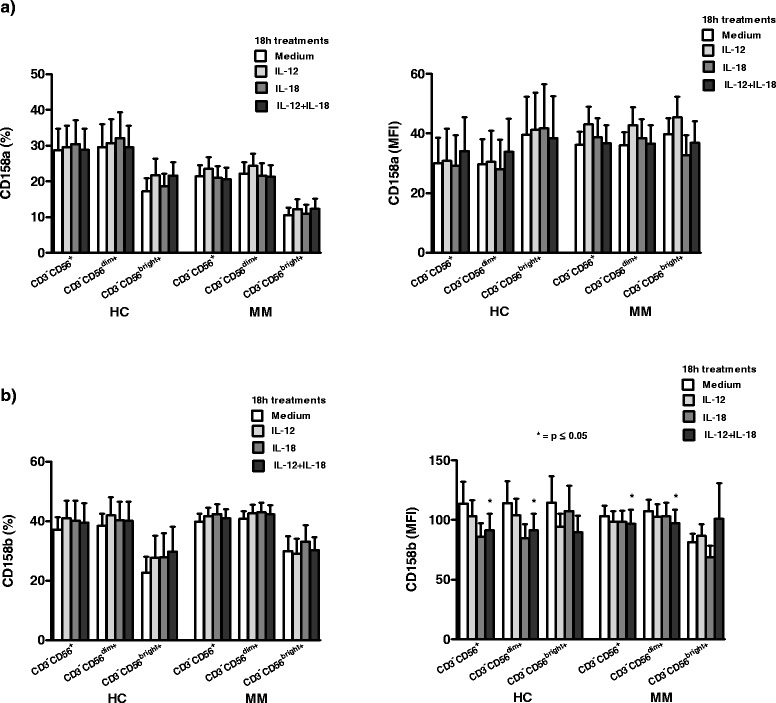
The effect of cytokines on CD158a and CD158b receptor expression on NK cell subsets. **a)** The percentage and mean fluorescence intensity (MFI) of CD158a KIR receptor do not change significantly (p > 0.05, Wilcoxon signed rank test) on CD3^−^CD56^+^ NK cells and their CD3^−^CD56^dim+^ and CD3^−^CD56^bright+^ subsets in healthy controls (HC) and metastatic melanoma (MM) patients after 18 h *in vitro* treatments of peripheral blood mononuclear cells (PBMC) with IL-12 or IL-18, alone, or with their combination; **b)** Contrary to the expression in percentage, MFI of the other inhibitory KIR, CD158b, decreases significantly (*p < 0.05, Wilcoxon signed rank test) on NK cells and the dim subset in both healthy controls (HC) and metastatic melanoma (MM) patients after 18 h *in vitro* treatment with IL-12 and IL-18 in combination. All results are obtained by Flow cytometry and are expressed as mean ± SE for 18 HC and 27 MM patients.

Analyzing the expression of KIR receptor, CD158a, on NK cells and their CD3^−^CD56^dim+^ and CD3^−^CD56^bright+^ subsets, we show that, compared to treatment in CM, the percentage and MFI of this receptor do not change significantly (p > 0.05, Wilcoxon signed rank test) after 18 h *in vitro* treatments with all investigated cytokines in HC, as well as in MM patients (Figure 5a).

Furthermore, contrary to the percentage expression of the other inhibitory KIR, CD158b, the MFI of this receptor decreases significantly (p < 0.05, Wilcoxon signed rank test) after 18 h *in vitro* treatment with combined IL-12 and IL-18 on NK cells and their dim subset in both HC and MM patients. IL-12 or IL-18 alone does not have any significant effect (p > 0.05, Wilcoxon signed rank test) on CD158b expression on NK cells and their subsets in HC and in MM patients (Figure 5b).

### The effect of investigated cytokines on CD25 receptor expression on NK cells and their subsets

**Figure 6:**
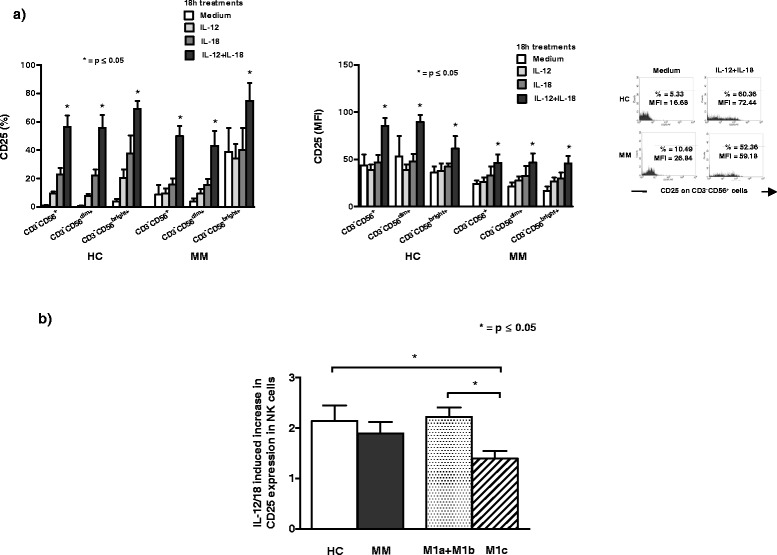
The effect of cytokines on CD25 receptor expression on NK cell subsets. **a)** The percentage and mean fluorescence intensity (MFI) of CD25 receptor increase significantly (*p < 0.05, Wilcoxon signed rank test) on CD3^−^CD56^+^NK cells and their CD3^−^CD56^dim+^ and CD3^−^CD56^bright+^ subsets in HC and MM patients after 18 h *in vitro* treatment with IL-12 and IL-18 in combination. Results are obtained by Flow cytometry; **b)** HC and MM patients in M1a + M1b group have significantly (*p < 0.05, Mann–Whitney exact test) higher increase in CD25 receptor expression on NK cells after *in vitro* treatment with combination of IL-12 and IL-18 compared to the increase in M1c group. Results are expressed in indexes calculated as the value of CD25 receptor expression after combined IL-12 and IL-18 treatment of each HC or MM patient devides with the value of this receptor expression after treatment in medium alone.

Analyzing the expression of CD25 receptor we show that both percentage and MFI of this receptor increase significantly (p < 0.05, Wilcoxon signed rank test) on NK cells and their dim and bright subsets in HC and MM patients after 18 h *in vitro* treatment with IL-12 and IL-18 in combination. IL-12 or IL-18 alone does not have any significant (p > 0.05, Wilcoxon signed rank test) effect on the expression of CD25 on NK cells and their subsets in HC and in MM patients (Figure 6a).

We also show that HC and MM patients that belong to M1a + M1b group have significantly (p < 0.05, Mann–Whitney exact test) higher increase in CD25 receptor expression on NK cells after *in vitro* treatment with combined IL-12 and IL-18 compared to the increase in M1c group (Figure 6b).

## Discussion

In this study we demonstrate that NK cell cytotoxicity and the expression of CD107a degranulation marker on NK cells and both their subsets in HC are high significantly augmented by the performed *in vitro* treatments with IL*-*12, alone, or IL*-*12 and IL*-*18 in combination that is in accord with the existing data [23-25]. It is well known that these cytokines upon binding to their high affinity receptors increase cytotoxicity of NK cells by inducing perforin, granzymes, FasL, TRAIL gene transcription either directly or indirectly by various STAT transcription factors [26].

The role of IL-18 in regulating NK cell activity in healthy individuals and especially in cancer patients is still very controversial. The lower effect of this cytokine on the increase of NK cell cytotoxicity in HC may be due to low, but detectable level of IL-1Rrp, a component of the IL-18R on native NK cells. The potentiation of NK cell cytotoxicity when IL-18 is combined with IL-12 may also be associated with this receptor as it has been shown that this receptor is up-regulated by IL-12 or IL-15 stimulation [27]. Furthermore, we show for the first time that in MM patients IL-18 alone does not have any significant effect on NK cell cytotoxic function and CD107a expression on NK cells and their subsets. It has been shown in new experimental melanoma models that IL-18 may convert conventional Kit^−^ into Kit^+^ NK cells that have immunosuppressive role [28].

Furthermore, as we show in this study that no one of investigated cytokine has the effect on the percentages, as well as the absolute values of NK cells and their dim and bright subsets in peripheral blood of HC and MM patients, the shown enhancement in NK cell cytotoxic function after *in vitro* cytokine treatment is not the consequence of increased number of investigated NK cells.

As it is well known that IL-12 and IL-18 have pronounced synergy in eliciting IFN-γ secretion from NK cells [29], we show that contrary to significantly induced MFI of IFN-γ in the entire population of cells, especially in CD56^bright+^ subset in HC, in MM patients only the bright subset produces significantly higher level of IFN-γ after combined IL-12 and IL-18 stimulation. Furthermore, we show that the level of this induction in the bright NK cell subset is significantly lower in MM patients compared to HC (data not shown). In this sense, we suggest that the other main effector function of NK cells, their immunoregulatory role, is suppressed in MM patients.

It is well known that in MM patients, the sites of metastases and serum levels of LDH are used to delineate these patients into three M categories: M1a, M1b, and M1c [20]. We show for the first time that the patients belonging to M1c category especially those patients that have distant metastasis in liver, as well as have increased level of serum LDH have significantly lower increase in NK cell cytotoxicity after *in vitro* treatment with combined IL-12 and IL-18 compared to the patients in M1a + M1b group that have metastases in distant skin, subcutaneous layer, lymph nodes and normal LDH serum values. These new data may suggest that contrary to MM patients that belong to M1c category with liver metastasis and elevated LDH, patients in M1a and M1b categories may be the target group for combined IL-12 and IL-18 treatment.

Furthermore, we show for the first time that MM patients that belong to M1c category have decreased expression of both IL-12 receptor subunits, IL-12R beta 1 and IL-12R beta 2, on their NK cells. It is well known that binding to IL-12R beta 1 and through IL-12R beta 2, IL-12 activates JAK-STAT signaling molecules, primarily STAT-4, and regulating perforin level enhances NK and T cell cytotoxicity [16]. Thus, decreased IL-12R beta 1 and IL-12R beta 2 expression on NK cells in M1c patients is in association with lower increase in pSTAT-4 and perforin expression in NK cells of these patients after IL-12 and combined IL-12 and IL-18 *in vitro* treatment shown in this study. In this sense, these new data may suggest, in M1c patients, the alteration in IL-12 signaling in their NK cells, as well as lower NK cell response to IL-12 or IL-12 and IL-18 in combination, the potential immunotherapy of MM patients.

In this study we also give results regarding the influence of this newer generation of cytokines on NK cell receptor expression.

We show for the first time that in MM patients IL-18 significantly decreases the expression of activating NKG2D receptor on NK cells and their cytotoxic dim subset. This result may explain inactiveness of IL-18 in increase of NK cell cytotoxicity in MM patients obtained in this study. Some clinical observations show that high serum levels of IL-18 in cancer patients may be associated with various NK cell defects [30,31], as well as that this cytokine may have the role in melanoma metastases [32]. So downregulation of NKG2D after IL-18 *in vitro* treatment shown in this study could facilitate escape of melanoma cells from NKG2D-mediated killing by NK cells.

Furthermore, we show for the first time that IL-18 decreases mRNA level of DAP10, NKG2D receptor signaling molecule, in PBMC of MM patients. Although DAP10 contributes not only to NK cells but also to CD8+ cytotoxic T lymphocytes (CTL)-mediated cytotoxicity via NKG2D receptor [33], in our experimental data we did not obtain the downregulation of NKG2D receptor on CTL after IL-18 *in vitro* PBMC treatment (data not shown). Therefore, decreased level of DAP10 in MM patients shown in this study could be associated with decreased NKG2D expression on NK cells and their dim subset in MM patients after IL-18 *in vitro* treatment. So far, there are no data regarding the effect of IL-12 and IL-18 on DAP10 level in NK cells and their subsets in MM patients.

As we show in this study that IL-12, IL-18 and their combination do not have any significant *in vitro* effect on DNAM-1 expression on NK cells and their subsets in HC, as well as in MM patients it is possible that DNAM-1 receptor expression on NK cells is not only the cytokine but also experimental conditions-dependent that is in accord with a few data in literature [34,35].

Furthermore, according to this study, IL-12 alone or in combination with IL-18 significantly increases CD161 expression on NK cells and their subsets only in HC. It is well known that the expression of CD161 is upregulated specifically by IL-12 [36]. The new results obtained in this study show that IL-12-induced CD161 activating receptor upregulation on NK cells and their subsets in HC, could be associated with this cytokine-increased NK cell cytotoxicity.

The data regarding the effect of cytokines on KIR expression in cancer patients are very rare [37]. It is now known that KIR downregulation could indicate a state of NK cell hyporesponsiveness [38]. In this sense, our new results regarding IL-12 and IL-18 *in vitro* effect on KIR expression could indicate that in HC and MM patients combined IL-12 and IL-18 treatment by decreasing CD158b receptor MFI on NK cells and cytotoxic dim subset regulates NK cell responsiveness and effector function. On the other hand, the observations obtained in this study may suggest the existence of cytokine-specific mechanisms of NK cell receptor expression regulation that is important in the maintenance of NK cell cytolytic machinery.

In this study we show for the first time that only combined IL-12 and IL-18 treatment significantly increases CD25, α chain of IL-2 receptor, on NK cells and both their subsets in HC, as well as in MM patients resulting in a constitution of functional high-affinity IL-2R (IL-2Rαβγ). However, we show that HC and MM patients that belong to M1a + M1b group have significantly higher increase in CD25 receptor expression after *in vitro* combined IL-12 and IL-18 treatment compared to the patients in M1c group. Recent data regarding mice *in vivo* and human healthy *in vitro* models show that IL-12, IL-15 and IL-18 preactivated NK cells express high level of CD25, proliferate rapidly and have enhanced effector functions especially IFN-γ production after stimulation with IL-2 [39]. Furthermore, these NK cells and their subsets are long-lived and might have memory-like properties [40,41]. Our new results suggest that adoptive transfer of IL-12 and IL-18 preactivated NK cells simultaneously treated with exogenous low dose of IL-2 may have an important place in immunotherapy of metastatic melanoma patients especially those patients belonging to M1a and M1b categories that have better survival outcome and prognosis.

## Conclusions

In this study we give insight into novel activation mechanisms of NK cells in HC and MM patients that underlie the effects of cytokines, IL-12, IL-18 and their combination. There are rare data about the role of these cytokines in the regulation of NK cell effector functions and the expression of various activating and inhibitory receptors on these cells and their dim and bright subsets in MM patients. Based on our new results, we suggest that *in vitro* treatment with IL-12 and IL-18, in combination, compared to IL-12 and IL-18 treatment, alone, have a prominent effect on NK cell cytotoxicity, IFN-γ production and the expression of CD25 receptor on NK cells in MM patients and especially in HC. Furthermore, MM patients that belong to M1c group especially those patients that have metastasis in liver and increased LDH serum values have significantly lower increase in NK cell cytotoxicity after *in vitro* treatment with IL-12 and IL-18 in combination compared to the patients that belong to M1a + M1b group. These results could be explained by decreased IL-12R beta 1 and IL-12R beta 2 expression on NK cells in M1c patients that is in association with lower increase in pSTAT-4 and perforin expression in NK cells of these patients after IL-12 and combined IL-12 and IL-18 *in vitro* treatment found for the first time in this study. So therapeutic strategies based on the adoptive transfer of IL-12 and IL-18 in combination preactivated NK cells might have an important place in immunotherapy of metastatic melanoma patients with better survival outcome and prognosis.

## Abbreviations

IL: Interleukin
IFN: Interferon
NKG2D: Natural killer group 2, member D
STAT: Signal tranducers and activators of transcription
MHC: Major histocompatibility complex
RT-PCR: Reverse transcriptase-polymerase chain reaction
FITC: Fluorescein isothiocyanate
PE: Phycoerythrin
